# Enhanced attenuation of chikungunya vaccines expressing antiviral cytokines

**DOI:** 10.1038/s41541-024-00843-x

**Published:** 2024-03-12

**Authors:** Christina Chuong, Chelsea Cereghino, Pallavi Rai, Tyler A. Bates, Megan Oberer, James Weger-Lucarelli

**Affiliations:** 1 Department of Biomedical Sciences and Pathobiology, Virginia Tech, VA-MD Regional College of Veterinary Medicine, Blacksburg, VA USA; 2 Center for Emerging, Zoonotic, and Arthropod-borne Pathogens, Virginia Tech, Blacksburg, VA USA

**Keywords:** Live attenuated vaccines, Alphaviruses, Viral infection

## Abstract

Alphaviruses are vector-borne, medically relevant, positive-stranded RNA viruses that cause disease in animals and humans worldwide. Of this group, chikungunya virus (CHIKV) is the most significant human pathogen, responsible for generating millions of infections leading to severe febrile illness and debilitating chronic joint pain. Currently, there are limited treatments to protect against alphavirus disease; thus, there is a tremendous need to generate safe and effective vaccines. Live-attenuated vaccines (LAVs) are cost-effective and potent immunization strategies capable of generating long-term protection in a single dose. However, LAVs often produce systemic viral replication, which can lead to unwanted post-vaccination side effects and pose a risk of reversion to a pathogenic phenotype and transmission to mosquitoes. Here, we utilized a chimeric infectious clone of CHIKV engineered with the domain C of the E2 gene of Semliki Forest virus (SFV) to express IFNγ and IL-21—two potent antiviral and immunomodulatory cytokines—in order to improve the LAV’s attenuation while maintaining immunogenicity. The IFNγ- and IL-21-expressing vaccine candidates were stable during passage and significantly attenuated post-vaccination, as mice experienced reduced footpad swelling with minimal systemic replication and dissemination capacity compared to the parental vaccine. Additionally, these candidates provided complete protection to mice challenged with WT CHIKV. Our dual attenuation strategy represents an innovative way to generate safe and effective alphavirus vaccines that could be applied to other viruses.

## Introduction

Chikungunya virus (CHIKV; Genus *Alphavirus*) is a mosquito-borne virus that has become a significant global public health threat. Since 2005, there have been over ten million reported CHIKV infections, mainly throughout the tropical and subtropical regions of Africa and Asia^1^. Millions of cases were reported in the Americas following CHIKV’s introduction in 2013, with small outbreaks also occurring in the US and Europe^2,3^. CHIKV resurged in the Americas in 2023, causing ~350,000 disease cases and spreading into new countries^4^. The recurrence of CHIKV outbreaks and its potential to become endemic in the United States warn us about similar viruses like Mayaro virus (MAYV) and Ross River virus (RRV); these viruses have recently caused multiple localized outbreaks, and their global spread may be facilitated by factors such as increased international travel, economic development, and climate-induced changes in mosquito vector distribution^5–9^. Therefore, CHIKV provides valuable insights into the potential emergence of other alphaviruses and serves as a model for developing essential preventative measures.

Infection with CHIKV leads to a severe febrile illness characterized by joint pain and swelling. Approximately 50% of people experience chronic joint pain that can persist for months or even years^10–14^. These enduring symptoms have significant adverse economic and social repercussions^15,16^. Currently, the management of chikungunya disease relies on non-specific nonsalicylate analgesics or non-steroidal anti-inflammatory drugs (NSAIDs), which exhibit varying levels of clinical effectiveness and do not prevent transmission^17^. There is only one recently approved vaccine and no therapeutics are available for preventing or treating alphavirus disease in humans. Therefore, there is an urgent need to further identify and implement effective treatments to reduce the impact of CHIKV in endemic regions, curbing its further spread and establishing a strategy to combat similar alphaviruses.

Various vaccine strategies for CHIKV are currently being explored: inactivated^18^, subunit^19,20^, VLP^21,22^, viral vectored^23,24^, mRNA vaccines^25^, and live-attenuated vaccines (LAVs). LAVs are considered the gold standard for viral vaccines because they are highly immunogenic and generally cost-effective while often providing robust protection from a single dose^26^. The effectiveness of a single-dose vaccine is particularly important in developing countries with limited resources, likely leading to higher immunization rates^27–29^. However, LAVs often have safety concerns, including adverse events and high vaccine virus replication that can lead to reversion to a pathogenic phenotype or mosquito transmission. For example, CHIKV 181/25, a LAV developed by the United States Army^30^, previously reached phase II clinical trials but caused adverse events in 8% of participants. Additionally, 181/25 had a high risk of reversion since its attenuation was only driven by two point mutations^31^. Another LAV, VLA1553, has recently been approved by the FDA and has shown encouraging immunogenic and protective qualities^32^. However, it has also been associated with footpad swelling in immunocompetent mice^33^ and systemic viral replication in mice, non-human primates, and humans^34,35^. Furthermore, > 60% of patients experienced systemic adverse events, including myalgia and arthralgia, in three different vaccine lots in a recent phase III clinical trial^36,37^.

To generate a safer CHIK LAV candidate, we combined two molecular attenuation mechanisms into a single vaccine construct. As the basis of our platform, we used a previously constructed chimera between CHIKV and its close relative Semliki Forest virus (SFV), where we replaced the domain C region of CHIKV’s receptor-binding protein, E2, with the corresponding region from SFV (CHIKV-SFV/DomC). CHIKV-SFV/DomC was highly attenuated in sensitive type I interferon signaling deficient mice (IFNAR^−/−^), and we observed significant reductions in footpad swelling, viremia, and replication in the brain when delivered intracranially^38^. Mosquito transmission was also significantly impaired in this chimera, which is critical to prevent vaccine virus escape^39^. However, given residual virus replication in IFNAR^−/−^ mice, we sought to further attenuate CHIKV-SFV/DomC.

Our secondary method of attenuation was the integration of different cytokines into the viral genome to be expressed constitutively during viral replication. Cytokines are critical immune signaling molecules responsible for a variety of activities, including activation, proliferation and expansion of leukocytes, recruitment of cells to areas of infection, and the activation of antiviral pathways. The utility of cytokines to stimulate vaccines as adjuvants has been proposed to elicit specific and robust immunity^40^. For example, several cancer vaccines have used IL-2, IL-15^41,42^, IL-21^43–46^, interferon-gamma (IFNγ)^47^, or granulocyte-macrophage colony-stimulating factor (GM-CSF), and have been tested in clinical trials; the latter candidate, a herpes virus expressing GM-CSF, received FDA approval for treatment of melanoma^48^. Furthermore, cytokine expression has been used to attenuate vaccinia virus^49,50^ and coxsackievirus B3^51^ using IL-12 and IFNγ. Thus, we engineered CHIKV-SFV/DomC to express IFNγ and IL-21, pleiotropic cytokines that regulate both innate and adaptive immune responses, contributing to robust antibody production and stimulating antiviral defenses^52^.

We hypothesized that the expression of these cytokines would attenuate LAV replication while maintaining or boosting neutralizing antibody responses. Following vaccination of immunocompetent or immunodeficient mice, both candidates demonstrated significantly reduced viral replication, dissemination, and footpad swelling compared to the non-cytokine-expressing control and CHIKV 181/25. Neutralizing antibody levels were increased in immunodeficient mice vaccinated with the IL-21-expressing virus but decreased in IFNγ-expressing LAV-vaccinated mice, as compared to CHIKV-SFV/DomC. Importantly, both vaccines maintained full protection against wild-type CHIKV challenge in immunocompetent and immunocompromised mice. This platform represents a promising strategy to generate protective vaccines with enhanced safety profiles and can serve as a basis for future RNA virus vaccines.

## Results

### Production and validation of cytokine-expressing vaccine candidates

**Fig. 1:**
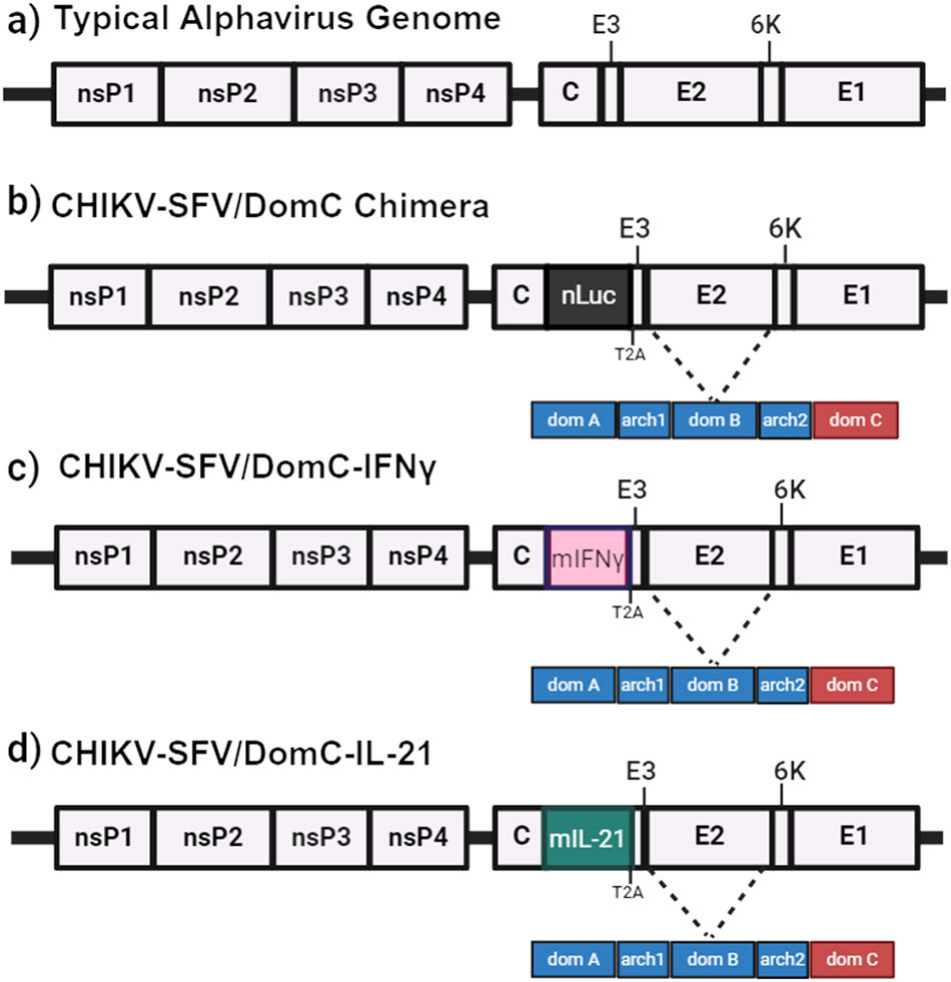
Genome organization of chimeric cytokine-expressing vaccines. Genome schematic of a (**a**) standard alphavirus genome, (**b**) the chimeric CHIKV genome with SFV domC (in red) expressing a nanoluc (nLuc) reporter gene, (**c**) the mouse IFNγ-expressing CHIKV/SFV chimera, and (**d**) the mouse IL-21-expressing CHIKV/SFV chimera. T2A refers to the Thosea asigna virus 2A self-cleaving peptide, which cleaves the foreign protein away from the viral envelope proteins.

We previously described a CHIKV/SFV chimera that replaces the E2 domain C region of CHIKV with the corresponding domain from SFV (Fig. 1a, b)^38,39^. This CHIKV-SFV/DomC chimera expresses nanoluciferase (*nLuc)* between the capsid gene and a Thosea asigna virus 2A-like self-cleaving peptide (T2A) and was attenuated in immunocompetent and immunocompromised mice, as well as in *Aedes aegypti* mosquitoes^38^. However, since CHIKV-SFV/DomC still produced viremia in IFNAR^−/−^ mice, we sought an additional means of attenuation. To that end, we replaced the *nLuc* gene with murine genes for *IFNγ* (Fig. 1c) or *IL-21* (Fig. 1d), two cytokines with potent stimulating activity for B and T cells and known antiviral activities^52,53^, hypothesizing that their expression would attenuate vaccine virus replication while maintaining or improving immunogenicity. Sequence analysis confirmed the correct insertion of each cytokine and both viruses were successfully rescued in HEK293T cells. Titers for CHIKV-SFV/DomC, CHIKV-SFV/DomC-IFNγ, and CHIKV-SFV/DomC-IL-21 collected after 48 h post-transfection were 7.5 log_10_ PFU/mL, 7.6 log_10_ PFU/mL, and 6.9 log_10_ PFU/mL, respectively.

**Fig. 2:**
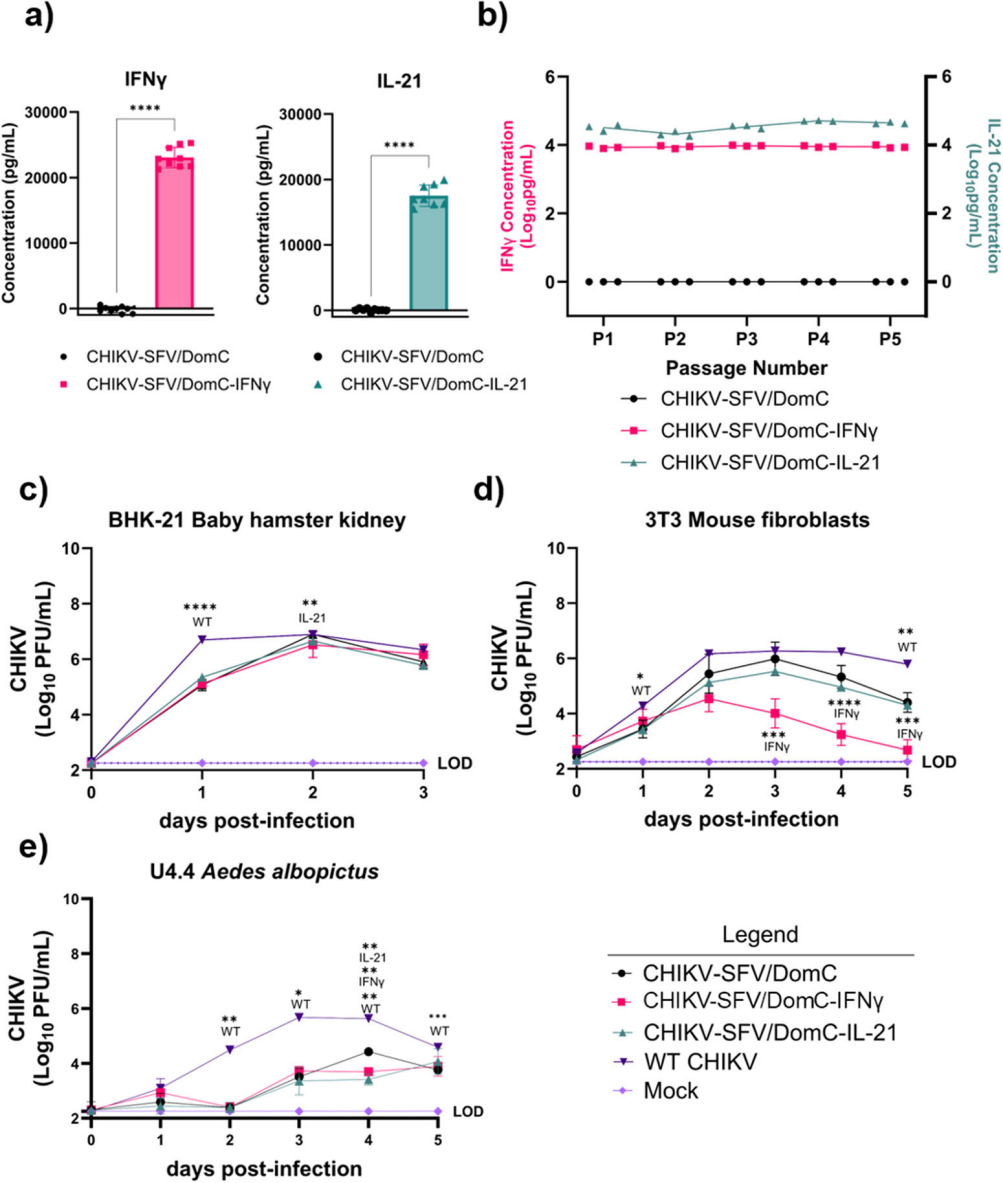
In vitro vaccine characterization. The concentration of mouse interferon-gamma (IFNγ) or mouse interleukin-21 (IL-21) (**a**) with respect to CHIKV-SFV/DomC was assessed by ELISA in viral supernatants rescued from HEK293T cells. Genetic stability of each cytokine was assessed by measuring the (**b**) concentration of IFNγ or IL-21 in CHIKV-SFV/DomC-IFNγ or CHIKV-SFV/DomC-IL-21, respectively, after viral passaging in Vero cells. The line for CHIKV-SFV/DomC represents values for both cytokines as values were undetectable for either cytokine. Each datapoint represents an independent passage series. Viral kinetics of all vaccine strains and WT CHIKV in (**c**) baby hamster kidney (BHK-21), (**d**) 3T3 mouse fibroblasts, and (**e**) U4.4 mosquito cells after infection at a multiplicity of infection of 0.01 PFU/cell. Data are shown as two biological replicates conducted in triplicate for ELISAs with statistical comparisons analyzed through unpaired *t*-tests. Concentrations were extrapolated from standard curves for each cytokine. Growth curve data is reported from two biological replicates for mammalian cells and one biological replicate for mosquito cells conducted in triplicates per group. Statistical comparisons with respect to the parental CHIKV-SFV/DomC were done using a mixed-effects model with Dunnett’s multiple comparisons test *P ≤ 0.05, **P ≤ 0.01, ***P ≤ 0.001, ****P ≤ 0.0001.

ELISAs were performed on viral stocks to quantify cytokine concentrations (Fig. 2a). IFNγ and IL-21 were significantly increased in CHIKV-SFV/DomC-IFNγ and CHIKV-SFV/DomC-IL-21 stocks, respectively, compared to CHIKV-SFV/DomC (*p* < 0.0001). To assess the stability of cytokine production, all viruses were serially passaged five times in the vaccine manufacturing approved Vero cell line. Consistent production of IFNγ and IL-21 was observed in CHIKV-SFV/DomC-IFNγ and CHIKV-SFV/DomC-Il-21, respectively, over each passage in contrast to CHIKV-SFV/DomC (Fig. 2b), indicating stable, high-level expression of each cytokine.

To assess the in vitro growth of each virus, we performed growth curves in several mammalian and mosquito cell lines. Initially, we used baby hamster kidney cells (BHK-21 clone 13), a highly susceptible cell line due to impaired type I IFN responses, to assess any baseline differences in viral replication between CHIKV-SFV/DomC and the cytokine-expressing vaccine candidates (Fig. 2c). All vaccine candidates replicated to significantly lower titers compared to WT CHIKV 1-day post-infection (dpi). By 2 dpi all viral titers had peaked, with CHIKV-SFV/DomC-IL-21 demonstrating lower viral titers compared to CHIKV-SFV/DomC (*p* = 0.0038).

Since the vaccines expressed mouse cytokines, we also infected mouse fibroblast 3T3 cells, an immunocompetent cell line representative of a likely target of infection following vaccination (Fig. 2d). WT CHIKV replicated to significantly higher titers compared to CHIKV-SFV/DomC (p_1dpi_ = 0.0118, p_5dpi_ = 0.002) and the cytokine-expressing candidates. CHIKV-SFV/DomC-IL-21 replicated similarly to CHIKV-SFV/DomC. In contrast, CHIKV-SFV/DomC-IFNγ replication was significantly attenuated with growth kinetics peaking at 2 dpi followed by a significant reduction compared to CHIKV-SFV/DomC by 3 dpi (*p* = 0.0004) and at 4–5 dpi (*p* < 0.0001).

Lastly, to assess replication of these vaccine candidates in mosquito cells, we infected U4.4 cells, an RNAi-competent *Aedes albopictus* cell line (Fig. 2e). CHIKV-SFV/DomC and cytokine-expressing candidates exhibited significantly decreased replication compared to WT CHIKV at all days after 1 dpi. Both CHIKV-SFV/DomC-IFNγ (*p* = 0.0087) and CHIKV-SFV/DomC-IL-21 (*p* = 0.0086) replicated similarly to CHIKV-SFV/DomC but with a significant attenuation observed at 4 dpi.

### Cytokine-expressing vaccine candidates protect against CHIK disease and virus replication in immunocompetent C57BL/6 mice

**Fig. 3:**
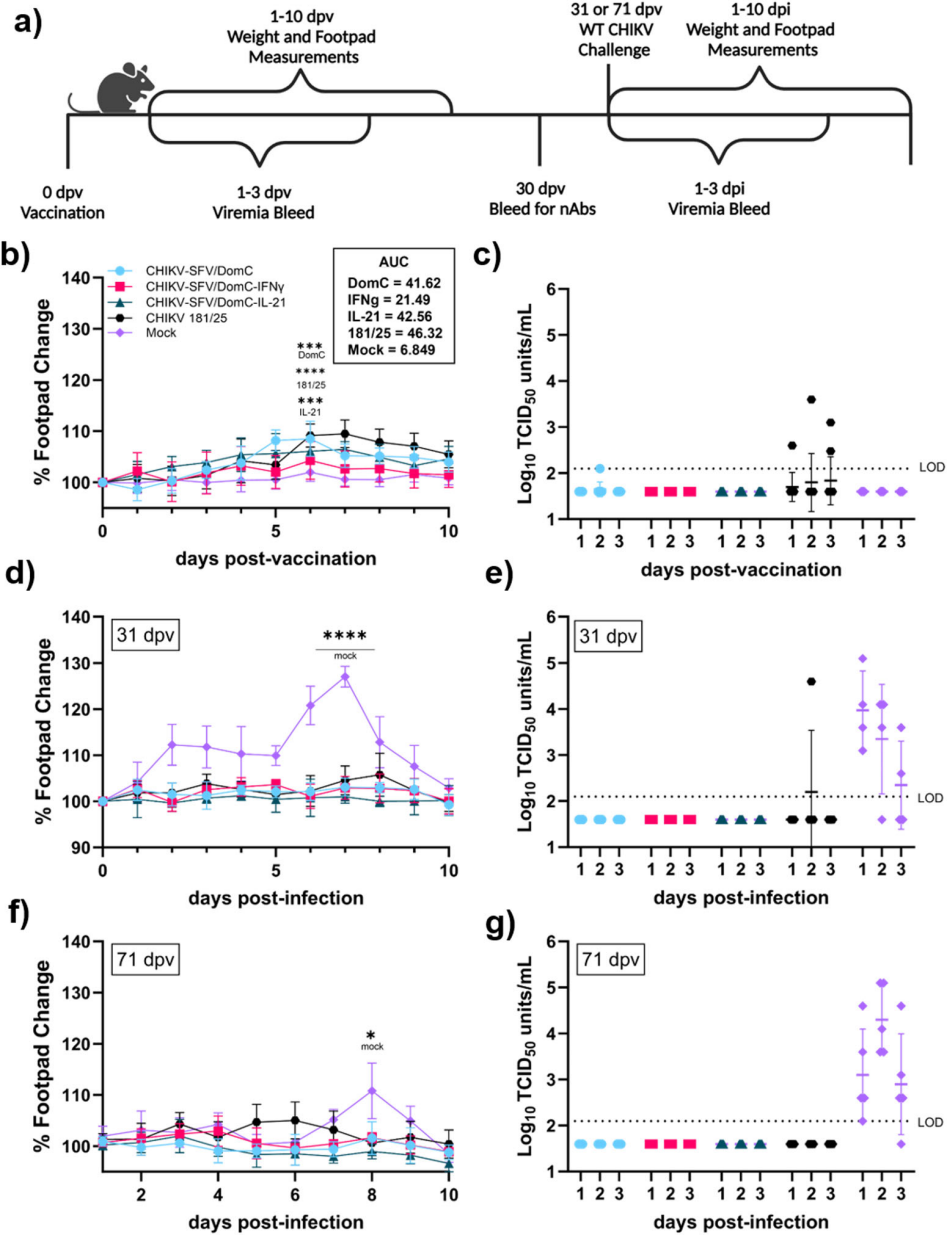
Vaccination and challenge of immunocompetent C57BL/6 mice. **a** Four-week-old male C57BL/6 mice (*n* = 5 per group) were inoculated via the left hind footpad with 10^4^ PFU of vaccine candidate or mock-infected with viral diluent. **b** Footpad swelling was measured daily and analyzed by area under the curve (AUC) analysis. **c** Mice were bled 3 days post-vaccination (dpv) to measure viremia by TCID50. Mice were challenged with 10^3^ PFU of WT CHIKV via the footpad 31 or 71 dpv with (**d**, **f**) footpad swelling monitored for each group, respectively. Mice were bled up to 3 days post-challenge to measure viremia as done previously (**e**, **g**). Post-vaccination data comprise of two independent experiments and one independent experiment for short- and longer-term challenges, respectively. Bars indicate standard deviations and dotted lines represent the limit of detection. Statistical comparisons were made by 2-way ANOVA, with Dunnett’s multiple comparisons test for footpad swelling *P ≤ 0.05, **P ≤ 0.01, ***P ≤ 0.001, ****P ≤ 0.0001.

Immunocompetent C57BL/6 mice can be used to model CHIKV infection as they are susceptible to mild disease, including biphasic ipsilateral footpad swelling and viremia^54,55^. To assess the effect of vaccine-mediated cytokine expression on vaccine safety and efficacy, we evaluated viral replication and disease following inoculation with each candidate in vivo. For an initial screen of our candidates, we used a validated 4-week-old male C57BL/6 mouse model, which are known to be susceptible to WT CHIKV infection^56–58^, to generate preliminary data on the relative susceptibility of our vaccines. The study design is presented in Fig. 3a. We inoculated the left hind footpad with 10^4^ PFU of CHIKV-SFV/DomC, CHIKV-SFV/DomC-IFNγ, CHIKV-SFV/DomC-IL-21, CHIKV 181/25 vaccine strain as a positive control, or viral diluent as a mock treatment. We measured footpad swelling daily as a marker of disease and collected blood for 3 days post-vaccination (dpv) to evaluate viral titers. As expected, we did not observe swelling in mock vaccinated mice. In comparison, all vaccinated mice except for the CHIKV-SFV/DomC-IFNγ group experienced significant footpad swelling peaking at 6 dpi compared to the mock-vaccinated mice, which was limited to less than a 10% increase in footpad width throughout the study (Fig. 3b). Total area under the curve (AUC) analyses demonstrate that CHIKV-SFV/DomC-IFNγ vaccinated mice had significantly decreased footpad swelling over 10 days compared to CHIKV-SFV/DomC vaccinated mice. In contrast, CHIKV 181/25 inoculated mice experienced greater footpad swelling with an initial delayed peak compared to the CHIKV-SFV/DomC vaccine.

Because the vaccine strains are attenuated and only caused mild disease, viremia was assessed by TCID_50_ through C6/36 amplification before transfer to Vero cells to increase the sensitivity to detect infectious virus, as previously described^54^ (Fig. 3c). CHIKV 181/25 was detected systemically on all 3 days post-vaccination. In contrast, CHIKV-SFV/DomC was observed at the limit of detection only at 2 dpv (2.1 log_10_TCID_50_/mL) in 1/10 mice. No systemic viral replication was detected in any mice vaccinated with either cytokine-expressing candidate at any time point post-vaccination. These data indicate that both our chimeric cytokine-expressing LAV candidates have limited capacity to generate viremia or footpad swelling in the WT C57BL/6 mouse model with an increased attenuation observed in the IFNγ-expressing vaccine candidate.

Since neutralizing antibodies (nAbs) are a common correlate of protection (CoP) for alphaviruses^33^, we measured nAbs 30 dpv by plaque reduction neutralization test (PRNT). We present the data as percent (%) neutralization of WT CHIKV over a series of serum dilutions (Supplementary Fig. 1a) and report the highest % neutralization achieved at the 1:20 serum dilution (Supplementary Fig. 1b). Compared to the mock-vaccinated group (11%), all vaccines had increased neutralization capacity in increasing order with CHIKV-SFV/DomC at 33.4% neutralization (*p* = 0.0049), CHIKV-SFV/DomC-IFNγ at 30.8% (*p* = 0.0152), CHIKV-SFV/DomC-IL-21 at 26.3% (*p* = 0.0830) and CHIKV 181/25 at 28.4% (*p* = 0.0385).

To assess the in vivo protective efficacy of the candidates, vaccinated mice were challenged with 10^3^ PFU of WT CHIKV SL15649, a typical viral load in the saliva of an infected mosquito^59^, 31 dpv. All vaccinated mice were protected from footpad swelling (Fig. 3d). Only mock-vaccinated mice exhibited the typical biphasic swelling pattern of CHIK disease in adult mice^54^. Initial swelling occurred at 2–3 dpi and peaked again at 7 dpi compared to CHIKV-SFV/DomC (*p* < 0.0001). Mice vaccinated with parental or cytokine-expressing candidates were fully protected from systemic viral replication (Fig. 3e). For CHIKV 181/25 vaccinated mice, 1/5 mice had detectable viremia 2 dpi. All mock-vaccinated mice became viremic.

To determine whether mice would maintain protection when challenged after an extended vaccination period, we infected a subset of vaccinated mice at 71 dpv. Mock-vaccinated mice were the only group to develop significant footpad swelling, which peaked at 8 dpi (Fig. 3f, *p* = 0.0036 compared to CHIKV-SFV/DomC). All mock-vaccinated mice became viremic at 1 and 2 dpi and 4/5 mice at 3 dpi. While the infection threshold for these studies was low, the results highlight the robust protection against systemic CHIKV replication and disease in immunocompetent C57BL/6 mice conferred by all CHIKV-SFV/DomC-based vaccine candidates.

### Cytokine-expressing vaccines are attenuated and have limited systemic replication in transiently immunocompromised C57BL/6 mice

Since WT C57BL/6 mice develop low viremia and mild footpad swelling following CHIKV infection, we sought to evaluate the safety and protection of our vaccine candidates in a more sensitive model of CHIKV infection^60^. CHIKV infection in humans is characterized by high viremia^61^ and systemic disease that is sometimes, but rarely, fatal^62^. CHIKV also robustly antagonizes type I IFN responses in humans but not in mice^59,63^. Thus, intending to use a model more representative of human CHIKV infection, we treated mice with a low dose (0.1 mg) of type I IFN receptor (IFNAR) antagonizing antibody MAR1–5A3 (herein referred to as MAR1) the day before infection. MAR1 has been used to establish widely accepted mouse models for Zika virus^64^, dengue virus^65^, Usutu virus^66^, and several alphaviruses^67–69^. We hypothesized that using a more susceptible model would reveal differences between vaccine candidates in terms of safety and protective efficacy. Additionally, by using a low dose of MAR1, we expected mice would be only slightly more susceptible to CHIKV; in contrast, CHIKV infection in fully immunocompromised mice such as IFNAR^−/−^ results in 100% mortality, which is an unrealistic model of CHIK disease^70,71^.

**Fig. 4:**
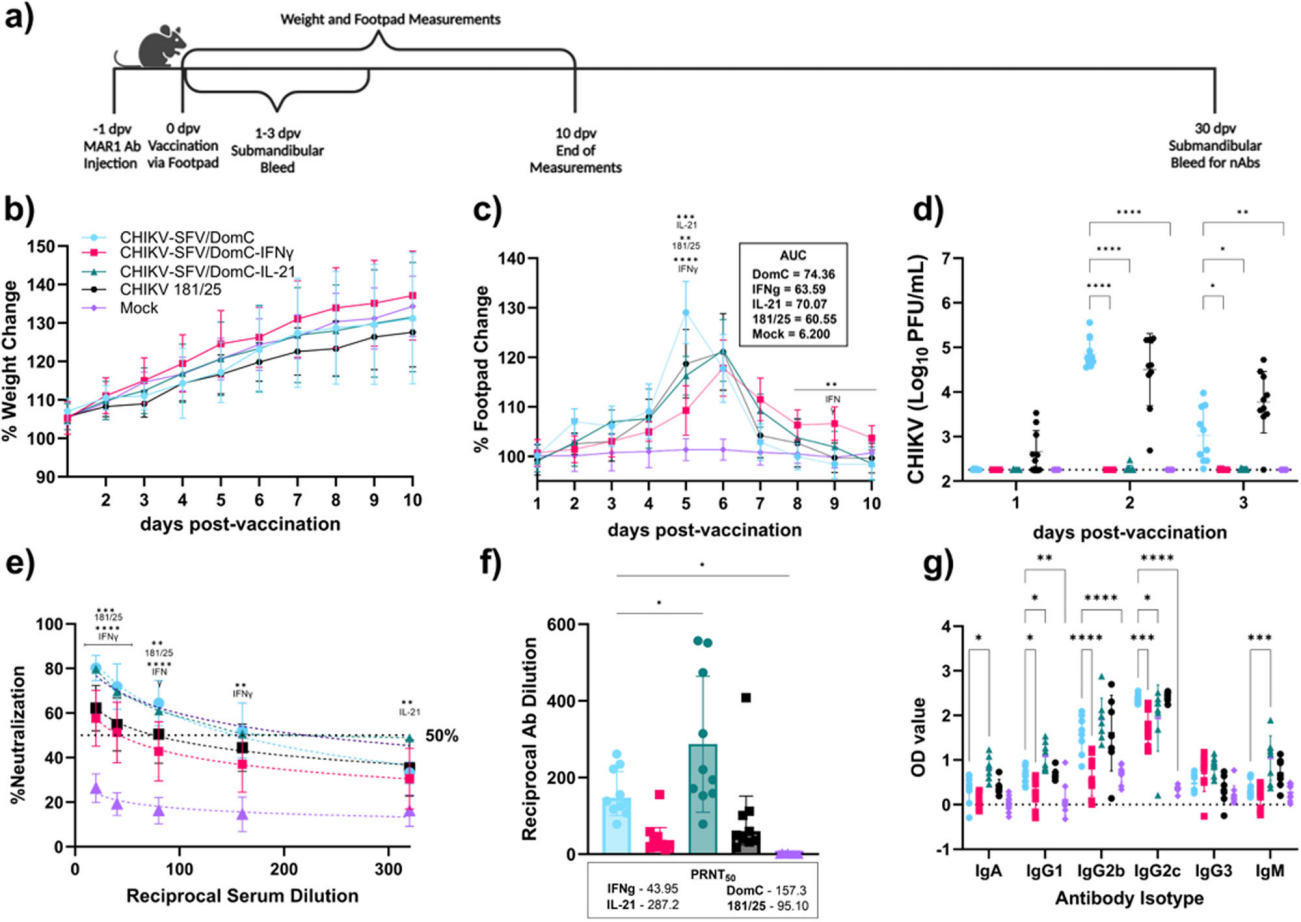
Vaccination of transiently immunocompromised C57BL/6 mice. **a** Mice (*n* = 10 per group) were inoculated via the footpad with 10^4^ PFU of vaccine candidate or mock-infected with viral diluent 24 h after MAR1 antibody (0.1 mg) treatment. **b** Weight and (**c**) footpad swelling were measured daily, with area under the curve (AOC) analyses presented, and (**d**) mice were bled daily for 3 days post-vaccination (dpv) to measure viremia by plaque assay. **e** Antibodies were assessed 30 dpv for percent WT CHIKV neutralization over a series of serum dilutions. **f** Mean PRNT_50_ values were determined through non-linear regression analysis and compared to CHIKV-SFV/DomC. **g** Isotype and antibody subclass responses after 30 dpv were measured by ELISA. Bars indicate standard deviations and dotted lines represent the limit of detection or 50% neutralization. Data is representative of two biological replicates of equal numbers of male and female mice. Statistical comparisons were made compared to CHIKV-SFV/DomC by either mixed-effects analysis or 2-way ANOVA with Dunnett’s multiple comparisons test for weights and footpad swelling, and viremia, %neutralization, and antibody isotyping, respectively. PRNT_50_ values were compared by one-way ANOVA with Dunnett’s multiple comparisons test; *P ≤ 0.05, **P ≤ 0.01, ***P ≤ 0.001, ****P ≤ 0.0001.

To assess safety and protection in this model, groups of 4-week-old male and female C57BL/6 were intraperitoneally given 0.1 mg of MAR1 (Fig. 4a). The age of the mice and dose of MAR1 were chosen based on previous literature to establish a highly susceptible but non-lethal CHIKV infection^67^. Additionally, since CHIKV infection is associated with a sex-dependent response^72,73^ we included both sexes in these studies. The following day, mice were inoculated in the left hind footpad with 10^4^ PFU of CHIKV-SFV/DomC, CHIKV-SFV/DomC-IFNγ, CHIKV-SFV/DomC-IL-21 or CHIKV 181/25 vaccine strain as a positive control. Mock-vaccinated mice were inoculated with viral diluent. We monitored health for 10 days following inoculation, as described in the immunocompetent mice studies. All vaccinated groups gained weight throughout the study, and no differences were observed in any chimeric vaccine candidate compared to mock-vaccinated mice (Fig. 4b). We observed a maximal increase in footpad swelling for CHIKV-SFV/DomC vaccinated mice at 5 dpv which was significantly increased compared to the maximum peak in CHIKV-SFV/DomC-IFNγ (*p* < 0.0001), CHIKV-SFV/DomC-IL-21(*p* = 0.0002), and CHIKV 181/25 vaccinated mice (*p* = 0.0087) (Fig. 4c). Additionally, AUC analyses showed an overall decreased total footpad swelling for both cytokine-expressing vaccine candidates compared to CHIKV-SFV/DomC. Similarly, viremia for both cytokine-expressing candidates was significantly reduced compared to CHIKV-SFV/DomC or 181/25 (Fig. 4d). CHIKV-SFV/DomC-IFNγ viremia was detectable in only 1/10 mice on 3 dpv with levels marginally above the limit of detection. CHIKV-SFV/DomC-IL-21 vaccinated mice also had limited viremia, with 3/10 mice having detectable viremia 2 dpv. These data demonstrate that both cytokine-expressing LAV candidates drastically reduce viremia in this more susceptible mouse model.

We also measured nAbs post-vaccination for each vaccine candidate by PRNT at 30 dpv. Serial dilutions of serum were mixed with WT CHIKV and reported as the percent reduction of plaques at each reciprocal dilution (Fig. 4e). Individual PRNT_50_ values, or the reciprocal serum dilution at which 50% of plaques are reduced, were generated from nonlinear regression of the percent neutralization data per mouse and compared to CHIKV-SFV/DomC (Fig. 4f). As expected, mock-vaccinated mice had no significant nAbs against CHIKV. All vaccinated mice neutralized CHIKV as shown by mean PRNT_50_ values in increasing order: CHIKV-SFV/DomC-IFNγ (43.95), CHIKV 181/25 (95.10), CHIKV-SFV/DomC (157.3) and CHIKV-SFV/DomC-IL-21 (287.2). CHIKV-SFV/DomC-IFNγ and 181/25 vaccinated mice PRNT_50_ values were not statistically different compared to CHIKV-SFV/DomC. In contrast, CHIKV-SFV/DomC-IL-21-vaccinated mice had significantly higher PRNT_50_ values compared to CHIKV-SFV/DomC. To assess differences in antibody isotypes and subclasses, we performed isotyping on the serum samples measuring levels of IgA, IgG1, IgG2b, IgG2c, IgG3, and IgM (Fig. 4g). All vaccinated mice induced high levels of IgG antibody subtypes compared to the mock-vaccinated mice. CHIKV-SFV/DomC mice generated significantly increased IgG1 (*p* = 0.0163) and IgG2c (*p* = 0.0006) titers compared to CHIKV-SFV/DomC-IFNγ and similar antibody titers to CHIKV 181/25 vaccinated mice. CHIKV-SFV/DomC-IL-21 vaccinated mice had significantly increased IgA, IgG1, IgG2b, and IgM, and significantly lower IgG2c antibodies compared to CHIKV-SFV/DomC. Altogether, CHIKV-SFV/DomC-IFNγ stimulated a lower antibody response with a slight shift towards a Th1-immune response ($\frac{{IgG}1}{{IgG}2c}\,$= 0.205) and CHIKV-SFV/DomC-IL-21 stimulated a stronger antibody response with a shift towards a Th2-immune response ($\frac{{IgG}1}{{IgG}2c}\,$= 0.499) based on the IgG1/IgG2c ratio compared to CHIKV-SFV/DomC ($\frac{{IgG}1}{{IgG}2c}\,$= 0.275). These antibody shifts are consistent with their respective cytokine profile for antibody class switching.

### Cytokine-expressing vaccine candidates protect against WT CHIKV challenge in transiently immunocompromised C57BL/6 mice

**Fig. 5:**
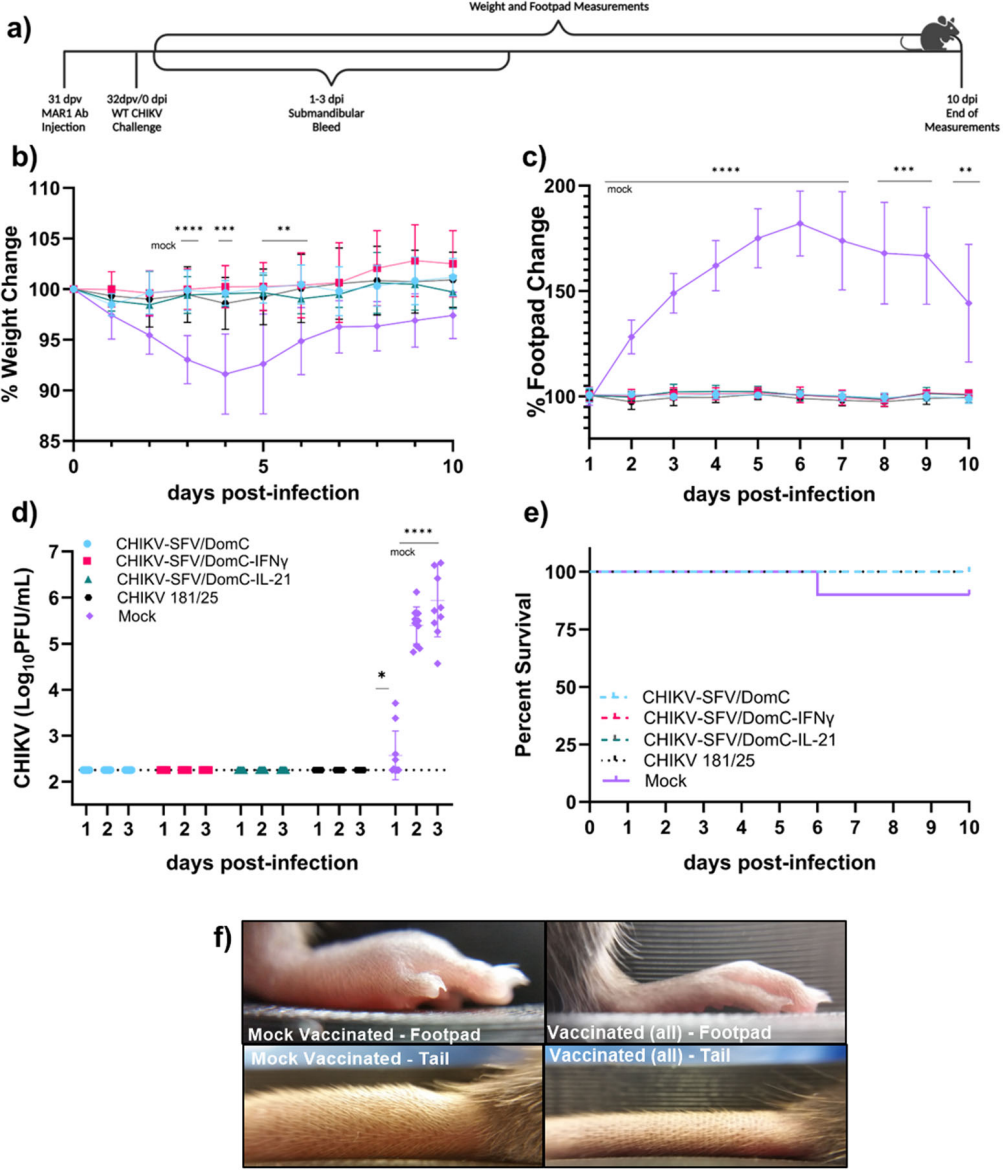
Wildtype CHIKV challenge of vaccinated immunocompromised C57BL/6 mice. **a** Vaccinated and mock-vaccinated mice (*n* = 10 per group) were inoculated via the footpad with 10^3^ PFU of WT CHIKV SL15649 32 days after vaccination after an additional MAR1 antibody (0.1 mg) treatment. **b** Weight and (**c**) footpad swelling were measured daily, and (**d**) mice were bled daily for 3 dpi to measure viremia by plaque assay. **e** Survival over the course of the study. **f** Representative images of footpad and tail swelling for mock-vaccinated compared to vaccinated mice are shown. Bars indicate standard deviations and dotted lines represent the limit of detection. Data is representative of two biological replicates using both male and female mice. Statistical comparisons were made compared to CHIKV-SFV/DomC by either mixed-effects analysis or 2-way ANOVA with Dunnett’s multiple comparisons test for weights and footpad swelling, and viremia, respectively; *P ≤ 0.05, **P ≤ 0.01, ***P ≤ 0.001, ****P ≤ 0.0001.

To evaluate the protective efficacy in mice vaccinated following MAR1 administration, we challenged the mice with 10^3^ PFU WT CHIKV following an additional treatment of 0.1 mg of MAR1 to produce a more potent challenge. The study design is presented in Fig. 5a. Following the challenge, all vaccinated mice remained at a steady weight (Fig. 5b), developed no footpad swelling (Fig. 5c), and had no detectable viremia (Fig. 5d). In contrast, mock-vaccinated mice lost considerable weight (*p* = 0.001) compared to CHIKV-SFV/DomC, experienced significant footpad swelling that peaked at 6 dpi, and generated high levels of viremia. Furthermore, 10% of mock-vaccinated mice experienced severe clinical disease requiring euthanasia by 6 dpi (Fig. 5e). Interestingly, mock-vaccinated mice also developed considerable swelling in the tail, which was not observed in any vaccinated groups (Fig. 5f). These data show that all vaccine candidates provided full protection against a virulent CHIKV challenge in a sensitive mouse model.

### Cytokine-expressing vaccine replication and dissemination are restricted in mice

**Fig. 6:**
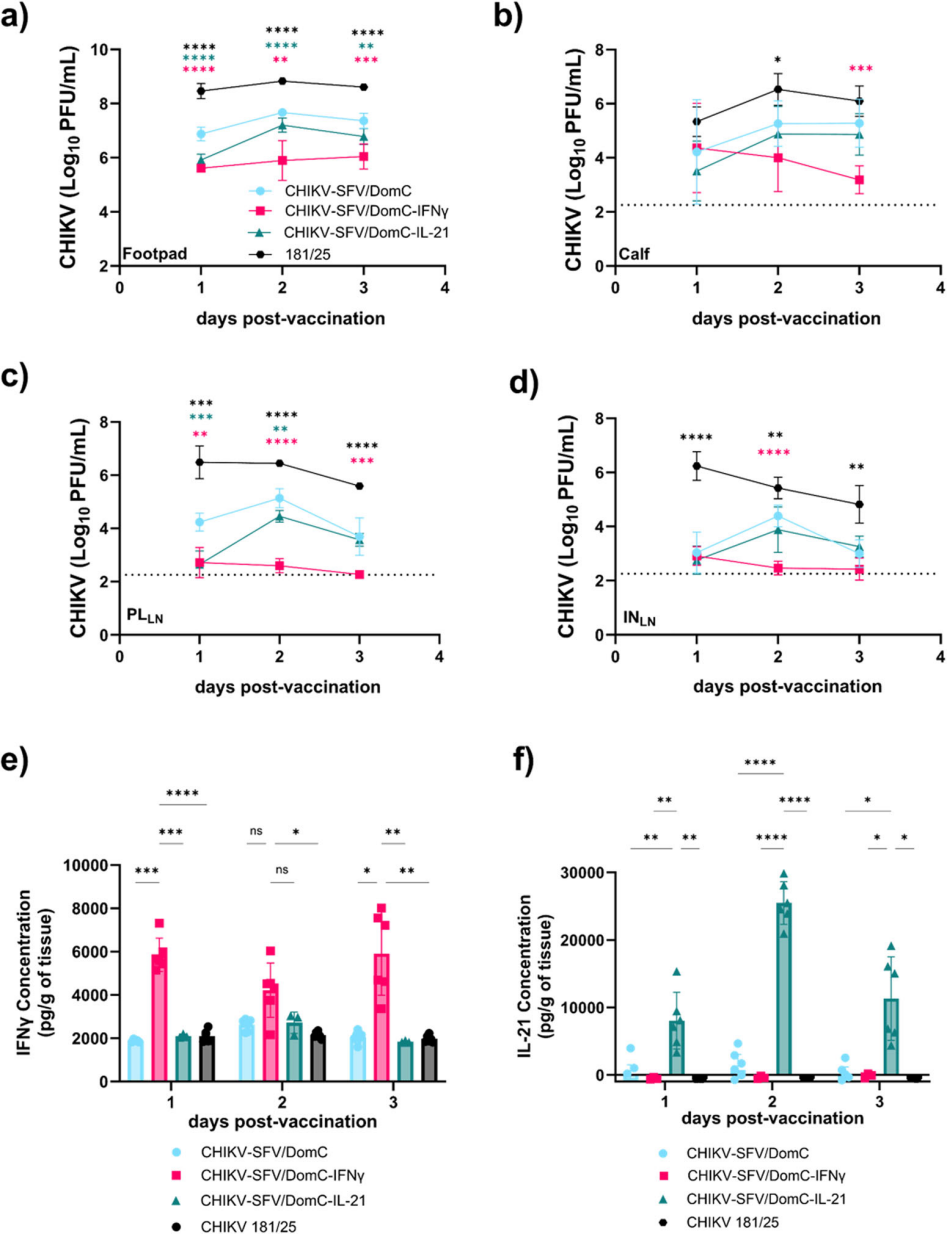
Viral dissemination and in vivo cytokine kinetics. Mice (*n* = 6–10 per group/day) were inoculated via the footpad with 10^4^ PFU of each vaccine candidate or mock-infected with viral diluent after MAR1 antibody (0.1 mg) treatment. **a** The inoculated footpad was dissected 1, 2, and 3 days post-vaccination (dpv) to assess viral titers by plaque assay. Viral titers were also assessed in the (**b**) calf tissues, (**c**) the popliteal lymph node [PL_LN_], and (**d**) the inguinal lymph node [IN_LN_]. Data was collected from two independent experiments. **e**, **f** Levels of interferon-gamma (IFNγ) and interleukin-21 (IL-21) were assessed in the inoculated footpads of vaccinated mice 1, 2, and 3 dpv. Bars indicate standard deviations and dotted lines represent the limit of detection. Statistical comparisons against CHIKV/SFV-DomC were performed using 2-way ANOVA with Dunnett’s or Šídák’s multiple comparisons test for viral titers and ELISAs, respectively. *P ≤ 0.05, **P ≤ 0.01, ***P ≤ 0.001, ****P ≤ 0.0001.

Toward understanding the attenuation of the cytokine-expressing vaccine candidates in the transiently immunocompromised model, we evaluated viral dissemination and replication kinetics in various tissues at 1, 2, and 3 dpv. High viral titers of CHIKV-SFV/DomC were observed in the footpad, peaking at 2 dpv (Fig. 6a). In contrast, both CHIKV-SFV/DomC-IL-21 and CHIKV-SFV/DomC-IFNγ replicated to significantly lower levels in all tissue samples tested compared to the parental control. Notably, CHIKV 181/25 replicated to significantly higher titers compared to CHIKV-SFV/DomC at all days post-vaccination.

Since viral replication was reduced at the site of inoculation, we further assessed viral titers in calf tissues and in the popliteal and inguinal lymph nodes of vaccinated mice to evaluate the impact on dissemination of each vaccine compared to CHIKV-SFV/DomC. In calf tissues, CHIKV-SFV/DomC inoculated mice had consistent viral titers from 1–3 dpv (Fig. 6b). There were no significant differences in CHIKV-SFV/DomC-IL-21 titers in the calf compared to CHIKV-SFV/DomC mice. In contrast, CHIKV-SFV/DomC-IFNγ replication began decreasing by 2 dpv and was significantly limited at 3 dpv. CHIKV 181/25 titers in the calf tissues were also highly elevated compared to the chimeric vaccines.

We observed a similar pattern in the popliteal (Fig. 6c) and inguinal lymph (Fig. 6d) nodes near the site of inoculation: CHIKV-SFV/DomC-IL-21 titers were reduced at early times point post-vaccination in the popliteal lymph node but not in the inguinal lymph node compared to CHIKV-SFV/DomC. CHIKV-SFV/DomC-IFNγ replication was significantly reduced in both lymph nodes, with titers just above our limit of detection (2.25 log_10_PFU/mL). CHIKV 181/25 had elevated titers in all tissues disseminating from the footpad. These data further demonstrate that vaccine virus-mediated expression of either IL-21 or IFNγ limits viral replication and dissemination in vivo and that our chimeric LAV strategy is more attenuated than 181/25.

To track and assess the impact of vaccine-mediated cytokine expression, we measured in vivo levels of both IFNγ (Fig. 6e) and IL-21 (Fig. 6f) in the footpads of each vaccinated candidate. Cytokine levels for each respective cytokine-expressing vaccine candidate were significantly elevated compared to the other viruses, indicating an appreciable effect from vaccine-mediated cytokine expression.

## Discussion

Outbreaks of emerging and zoonotic viruses such as CHIKV and other alphaviruses are occurring at increasing frequency^74^. Vaccination is a key approach to prevent disease; thus, effective alphavirus vaccines are crucial to avoid future pandemics. Ideally, this vaccine would be highly effective and safe. Additionally, a vaccine platform that could be adapted for similar viruses would be highly advantageous. LAVs are historically the most effective vaccines, including against arboviral diseases like yellow fever^26^. However, since these viruses are replication-competent, safety concerns remain, especially in immunocompromised people. Furthermore, typical LAVs attenuated by serial passage have the potential to revert to a pathogenic phenotype or escape into a natural transmission cycle since few mutations typically mediate their attenuation^75,76^. Thus, engineering robust molecular attenuation mechanisms into LAVs is a promising approach to predictably generate safe and effective LAVs for a broad range of pathogens.

Here, we engineered our previously described chimeric virus backbone to express one of two cytokines with potent and pleiotropic roles in innate and adaptive immunity, IFNγ or IL-21. Each candidate was capable of replicating to high titers in Vero cells and maintained stable production of each respective cytokine over five passages, indicating potential scale-up capacity without loss of cytokine expression. To assess efficacy, we evaluated the candidates in several mouse models of CHIKV infection in comparison to both the parental non-cytokine-expressing chimera and CHIKV 181/25, which was tested in phase II clinical trials but discontinued due to safety concerns. Cytokine-expressing vaccines had increased safety in vivo, showing significantly decreased footpad swelling and reduced viral replication and dissemination. Furthermore, both cytokine-expressing vaccines afforded full protection against WT CHIKV.

IFNγ is a pleiotropic cytokine capable of stimulating broad antiviral pathways as well as the proliferation and maturation of macrophages, T_h_1 CD4 T cells, and cytolytic CD8 T cells^77–80^. IFNγ has previously been expressed in recombinant viral vectors to limit replication of vaccinia virus and coxsackievirus B3^51,81^. It has also been used successfully as a post-exposure prophylaxis capable of preventing morbidity from smallpox^82^. Clinically, IFNγ has demonstrated T cell-enhancing properties to inhibit tumor proliferation in cancers such as multiple myeloma and melanoma^47,83^. While type I IFNs are typically associated with robust antiviral activity against alphaviruses, IFNγ also contributes to controlling alphavirus replication and spread. Specifically, mice defective in IFN α/β/γ receptor signaling had more severe disease and increased viremia after CHIKV infection compared to mice only defective in IFN α/β receptor signaling^84^. Our in vitro growth curves in 3T3 mouse fibroblasts showed a strong replication restriction for CHIKV-SFV/DomC-IFNγ, suggesting an antiviral effect mediated by cytokine expression. Thus, we hypothesized that vaccination in mice with CHIKV-SFV/DomC-IFNγ would lead to reduced replication and dissemination, and thus limit post-vaccination side effects. CHIKV-SFV/DomC-IFNγ vaccinated mice had significantly reduced viral replication, dissemination, and disease compared to CHIKV-SFV/DomC in MAR1-treated C57BL/6 mice. We then asked whether vaccine-expressed IFNγ would skew isotype switching, increasing levels of IgG2c, a potent antiviral subclass^85^. While there was a slight shift in the ratio of IgG1/IgG2c antibodies promoting a T_h_1-polarized response, overall levels of antibodies were decreased in CHIKV-SFV/DomC-IFNγ vaccinated mice, which may be due to its increased attenuation and more efficient control of vaccine replication. However, this did not impact the capacity to protect against WT CHIKV protection.

As a different mechanism of stimulating the immune system compared to IFNγ, we explored the potential of IL-21 to enhance attenuation and immunogenicity of CHIKV-SFV/DomC. IL-21 is normally produced by a variety of T cells including CD8, CD4, T_h_17, T_FH_, and natural killer (NK) T cells^86,87^ and plays a significant role in promoting B cell proliferation, maturation, and differentiation^88^. Particularly, IL-21 directly acts on B cells to control germinal center formation, regulating antibody production to enhance IgM, IgG, and IgA levels^87,89,90^. Additionally, in contrast to our IFNγ-expressing candidate, IL-21 is thought to “fine-tune” a T_h_2-associated immune response^91,92^. Finally, IL-21 is also known to contribute to antiviral responses through activation of various immune cells^52^. Thus, we hypothesized that IL-21 would act more specifically on effector cells impacting the landscape of protection through antibody production while also exerting an antiviral effect.

In contrast to our IFNγ-expressing candidate, the IL-21-expressing LAV did not replicate lower in vitro in a mouse fibroblast cell line. However, in vivo vaccination with CHIKV-SFV/DomC-IL-21 resulted in a significant reduction in viral replication compared to CHIKV-SFV/DomC, preventing systemic virus replication and reducing dissemination into local tissues. This reduction in replication was not as pronounced compared to CHIKV-SFV/DomC-IFNγ. However, the IL-21-expressing CHIKV vaccine significantly enhanced antibody levels of IgG1, IgG3, IgM, and IgA as well as generated an increased neutralizing antibody titer compared to all other vaccine candidates tested.

Given the differences observed between the two cytokine-expressing candidates, it would be interesting to explore the cross-protective capabilities of these vaccines against other alphaviruses. Alphavirus neutralizing antibodies primarily target the domain B region of the E2 glycoprotein and provide some broad cross-protective capabilities^67,68,93^. In our chimeric backbone, CHIKV-SFV/DomC, the E2 domain B is intact and has already been shown to protect against lethal challenges against both CHIKV and SFV^38^. IFNγ or IL-21 expression may possibly improve cross-protection against other alphaviruses.

Some limitations of our studies include the use of young C57BL/6 mice; future studies should vaccinate older mice to assess immunogenicity in mice of varying ages. While there were some improvements in post-vaccination footpad swelling in the IFNγ-expressing vaccine and all our vaccine candidates afforded protection against WT CHIKV, ultimately, the chimeric vaccines were too attenuated to show distinct cytokine-dependent activity. We used a vaccine dose of 10^4^ PFU, as previously used in clinical trials^94^, to assess protection here; however, an increased dose may be more effective. Further, while our long-term vaccine challenge in immunocompetent mice protected from viremia, we did not observe a strong disease outcome in the mock-vaccinated mice as measured by footpad swelling; thus, these studies should use a more robust challenge model in the future. Another limitation of our study was not characterizing other immune responses or specific subsets of CD4  and CD8  T cells or other lymphoid and myeloid cells that could have been stimulated by IFNγ or IL-21. Our goal was to first assess the viability of these vaccines in improving in vivo vaccination and protection outcomes as well as to assess immunity by measuring neutralizing antibodies. Future studies will interrogate the mechanistic pathways activated by our cytokine-expressing vaccines and systematically determine the correlates of vaccine protection.

In summary, we present an innovative molecular strategy that combines two distinct attenuation mechanisms to create a safer LAV that maintains full protection against virulent CHIKV challenge. The mechanism of attenuation of each candidate appears to be unique given the differences in replication kinetics in vitro and in vivo and warrants further investigation, including whether these cytokines have an increased combined therapeutic potential. Furthermore, these studies highlight the potential utility of using cytokine-expressing viruses to study how different immune components affect viral replication and disease outcomes. Our work establishes a promising platform for designing a new generation of effective LAVs with enhanced safety attributes.

## Methods

### Ethics statement

This study was carried out in accordance with the guidelines in the Guide for the Care and Use of Laboratory Animals of the National Institutes of Health. The research protocol was approved by the Institutional Animal Care and Use Committee (IACUC) of Virginia Tech. Mice were euthanized using CO_2_ followed by cervical dislocation according to the 2020 American Veterinary Medical Association (AVMA) guidelines.

### Cell culture and viruses

We maintained BHK-21 (ATCC CCL-10, hamster kidney fibroblast), Vero (ATCC CCL-81, African green monkey kidney epithelial), HEK293T (ATCC CRL-3216, human kidney epithelial), 3T3 (ATCC CRL-1658, mouse embryo fibroblast) at 37 °C in 5% CO_2_. U4.4, courtesy of Nathan Grubaugh (Yale University) cells were maintained at 28 °C in 5% CO_2_. All cell lines were grown in Dulbecco’s modified Eagle’s medium (DMEM) supplemented with 5% fetal bovine serum (FBS), 1% nonessential amino acids, and 0.1% gentamicin (herein referred to as DMEM-5). All CHIKV vaccine candidates were modified from a chimeric virus clone^39^ originally derived from a wild-type (WT) clone of strain SL-CK1^95^. WT CHIKV SL15649 clone-derived virus, courtesy of Mark Heise (the University of North Carolina at Chapel Hill), was used in challenge studies.

### Generation of CHIKV vaccine candidates

The construction of CHIKV-SFV/DomC, which expresses nanoluciferase (nLuc; Promega, WI, USA) was described previously^38^. Briefly, an infectious clone of CHIKV strain SL-CK1 was constructed using overlap extension PCR with a CMV promoter for viral rescue in mammalian cells. In the parental CHIKV/SFV-DomC virus, the reporter sequence for NanoLuc luciferase (nLuc) was engineered as a cleavable in-frame fusion between the capsid and E3 protein followed by a Thosea asigna virus (TaV) 2A-like protease sequence to fully release the nascent protein. Platinum SuperFi 2X master mix (ThermoFisher, MA, USA) was used to generate vector backbone segments around nLuc and cytokine fragments (Sino Biological, PA, USA) with the following primers: Forward 5’-AGAGTGGAGTCTTGCCATCCCAGTTATGAACGCTACACACTGCATCTTG-3’ and Reverse 5’- GCGGAAAAGGAGTCGCTGCCGCGCCGAGGGCAGAGGAA-3’, and Forward 5’-AGAGTGGAGTCTTGCCATCCCAGTTATGGAGAGGACCCTTGTCTGTC-3’ and Reverse 5’- AGATGATTCATCAGCATCTCTCCCGCGCCGAGGGCAGAGGAA-3’, for CHIKV-SFV/DomC-IFNγ and CHIKV-SFV/DomC-IL-21, respectively. Murine IL-21 and IFNγ cytokine fragment was individually assembled into the vector backbone using NEBuilder HiFi DNA Assembly Master Mix at a 1:2 (vector: insert) molar ratio to generate each vaccine candidate. Hifi assembly was treated with DpnI before electroporation into homemade NEB stable electrocompetent cells. Cells were then plated onto LB agar with 50 μg/mL carbenicillin and grown at 30 °C overnight. Single isolated colonies were grown in liquid cultures, and plasmids were extracted using the NucleoSpin Plasmid Transfection-grade kit (Macherey-Nagel, PA, USA). Insertion of each cytokine was confirmed with Sanger sequencing. The sequence for CHIKV-SFV/DomC has been submitted to NCBI’s GenBank under accession number OR782938. For virus rescue, plasmids for each vaccine candidate were transfected into HEK-293T cells using JetOPTIMUS (Polyplus, Illkirch, France) according to the manufacturer’s protocol. Viral supernatants (p0) were collected, aliquoted, and stored at −80 °C for us in all experiments.

### Virus and antibody titers

Plaque assays and plaque reduction neutralization tests (PRNTs) were performed in Vero cells as previously described in ref. 65. For mouse sera collected from immunocompetent C57BL/6 mice, viral titers were assessed by C6/36 pre-amplification followed by transfer to Vero cells as previously described in ref. 54. Briefly, C6/36 s were plated at 15,000 cells/well in 96 well plates. Serum from each mouse was serial diluted in Dulbecco’s modified Eagle’s medium (DMEM) supplemented with 2% fetal bovine serum (FBS) onto the cells and incubated for 4 days at 28 °C in 5% CO_2_. After incubation, C3/36 inoculates were transferred onto confluent 96-well plates of Vero cells and incubated 37 °C in 5% CO_2_ until visible CPE (3 days). Viral supernatants were decanted and cells were fixed with crystal violet. Viral titers for 50% tissue culture infectious dose (TCID50) were calculated utilizing the Spearman & Kärber algorithm.

### Enzyme-linked immunosorbent assays (ELISAs)

Cytokine expression was assessed using Bio-Techne’s DuoSet kits for mouse IFNγ (DY485–05) and IL-21 (DY594–05) according to the manufacturer’s protocol.

### Cell culture passages

All chimeric vaccines were passaged in Vero cells to assess viral replication capacity in a standard in vitro vaccine production cell line and to assess the genetic stability of the cytokines. Veros were grown to 80–90% confluency and then were infected at a multiplicity of infection (MOI) of 0.01. Following a 48 h incubation period, viral supernatants were collected and clarified before infection of a second passage for a total of 5 passages. Each passage was performed in triplicate wells and expanded independently. Cytokine levels of each passage for CHIKV-SFV/DomC, CHIKV-SFV/DomC-IFNγ, and CHIKV-SFV/DomC-IL-21 were assessed by ELISA.

### Viral growth kinetics

Twenty-four well plates were grown to 80–90% confluency for BHK-21 clone 13, 3T3 fibroblasts, and U4.4 cells. Cells were infected at a multiplicity of infection (MOI) of 0.01 PFU/cell for each vaccine candidate and assessed against the parental CHIKV-SFV/DomC and the original WT strain SL-CK1. Virus stocks were diluted in Roswell Park Memorial Institute medium (RPMI 1640) with 25 mM HEPES and 1% FBS (herein referred to as viral diluent). The viral inoculum was added to each well in triplicate, rocked, and incubated at 37 °C in 5% CO_2_ for 1 h. Back-titers of viral inocula were also assessed to confirm MOI. After adsorption, cells were washed 1–2 times with phosphate-buffered saline (PBS), and DMEM-5 was added to each well. A 0 h-time point was collected to assess baseline viral titers, and supernatants were harvested every 24 h until 50–75% CPE was observed. Samples were stored at −80 °C until titration by plaque assay.

### Animal studies

*Immunocompetent mice*. Four-week-old C57BL/6 mice were obtained from The Jackson Laboratory (ME, USA). Groups of male mice (*n* = 10 per group) were infected in the hind-left footpad with 10^4^ PFU of one of the CHIKV vaccine candidates or mock-infected with viral diluent. Mice were weighed and monitored daily for signs of disease. Footpad widths were measured daily using digital calipers. Mice were anesthetized using isoflurane and then bled via the maxillary vein each day for three days post-infection. Thirty-one- or seventy-one-days post-vaccination (dpv), five mice per group were infected with 10^3^ PFU of WT CHIKV and monitored as previously described.

*Immunocompromised mice*. Four-week-old C57BL/6 male and female mice (*n* = 5 per group/sex) were vaccinated with the candidates and challenged with WT CHIKV as earlier described; however, before viral inoculations, mice were intraperitoneally (i.p.) inoculated with 0.1 mg of MAR1–5A3, an anti-interferon-α/β receptor (IFNAR) antibody (Leinco Technologies, Fenton, MO)^96^. Euthanasia criteria included weight loss greater than 15% of initial body weight. Mice were bled through the maxillary vein 30 dpv to assess neutralizing antibodies. For viral dissemination studies, groups of male mice (*n* = 6–10, per group/day) were treated with 0.1 mg of MAR1–5A3 prior to vaccine inoculation as described above and were euthanized 1, 2, and 3 dpv to collect footpad and calf tissues, and popliteal and inguinal lymph nodes. Tissues were collected in 2 mL tubes with a metal bead, and viral diluent was added at a 10% weight/volume ratio. Tissues were homogenized with a Tissue Lyser II (Qiagen, Germantown, MD) at 25 freq/s for 2 min at a time until tissues were fully homogenized. After collection of clarified tissue supernatant, 1x DNA/RNA shield was added to the remaining footpad tissues and re-homogenized. RNA was extracted from the footpad with the Quick-RNA Miniprep Plus kit (Zymo Research, Irvine, CA, USA) per the manufacturer’s instructions.

### Statistical Analysis

Statistical comparisons were performed in Prism 9 (GraphPad, CA, USA). Unpaired t-tests and 2-way ANOVA with Dunnett’s multiple comparisons test were performed for ELISAs and growth curves, respectively. For animal weight and footpad swelling, statistical comparisons were made with a mixed-effects analysis with Dunnett’s multiple comparisons test. PRNT_50_ was calculated using nonlinear regression curve fitting and values were compared through ordinary one-way ANOVA. One-way ANOVA with Dunnett’s multiple comparisons test or unpaired t tests were used for comparisons between viral titers and gene expression with respect to mock treatment virus only conditions. Error bars indicate standard deviation.

### Reporting summary

Further information on research design is available in the Nature Research Reporting Summary linked to this article.

### Supplementary information

Reporting summary

Supplementary figure

## Data Availability

The sequence for the cytokine-expressing cDNA clone of CHIKV-SFV/DomC is available on GenBank (Accession no. OR782938). For additional data and figures, please contact the corresponding author at weger@vt.edu.
