# Author Correction: Enhanced attenuation of chikungunya vaccines expressing antiviral cytokines

**DOI:** 10.1038/s41541-024-00868-2

**Published:** 2024-04-16

**Authors:** Christina Chuong, Chelsea Cereghino, Pallavi Rai, Tyler A. Bates, Megan Oberer, James Weger-Lucarelli

**Affiliations:** 1 Department of Biomedical Sciences and Pathobiology, Virginia Tech, VA-MD Regional College of Veterinary Medicine, Blacksburg, VA USA; 2 Center for Emerging, Zoonotic, and Arthropod-borne Pathogens, Virginia Tech, Blacksburg, VA USA

**Keywords:** Live attenuated vaccines, Alphaviruses, Viral infection

Correction to: *npj Vaccines* 10.1038/s41541-024-00843-x, published online 12 March 2024

In this article, the author’s name Chelsea Cereghino was incorrectly written as Chelsea N. Cereghino. The original article has been corrected.

