# Supplementary figures and images for: Enhanced attenuation of chikungunya vaccines expressing antiviral cytokines
Source: NPJ Vaccines. 2024 Mar 12;9:59. doi: 10.1038/s41541-024-00843-x (PMC10933427; doi:10.1038/s41541-024-00843-x)

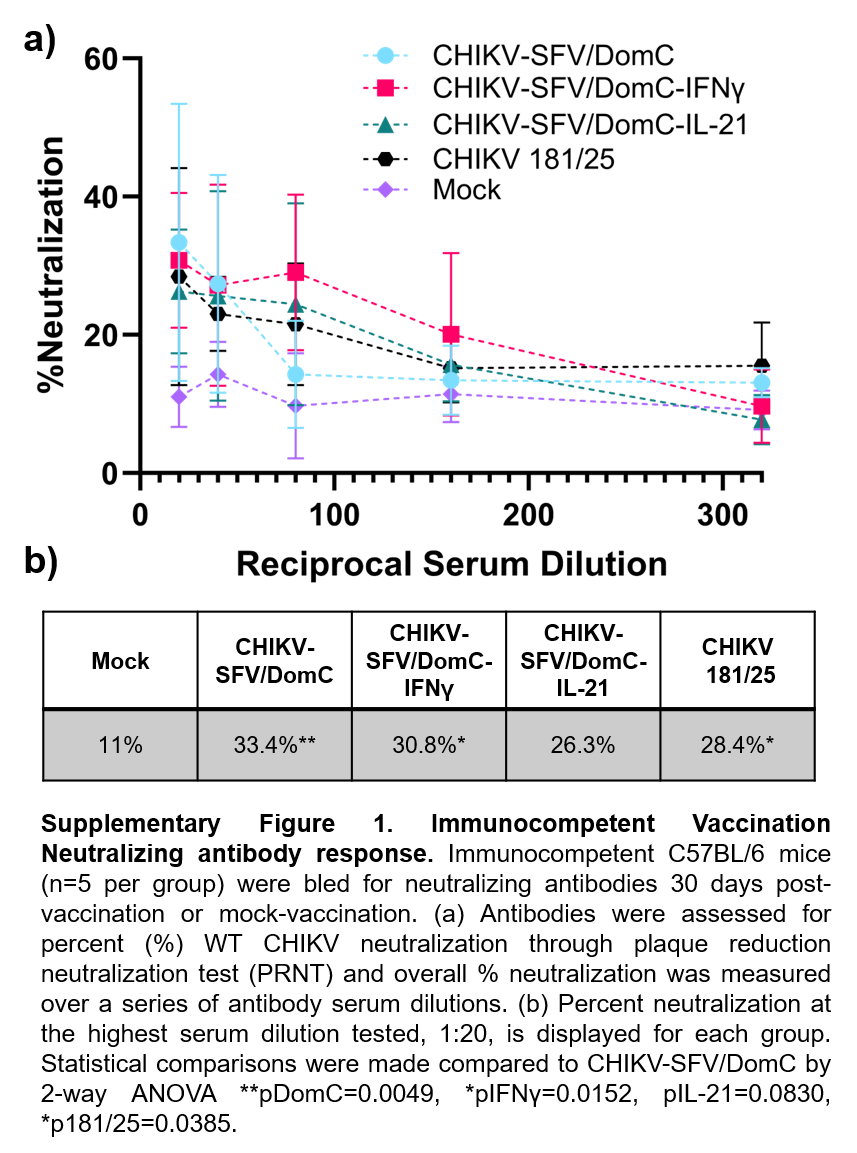

Supplement: Supplementary file 2 — Supplementary figure [file 41541_2024_843_MOESM2_ESM.tif]
